# Readmission rates and risk factors for readmission after transcatheter aortic valve replacement in patients with end-stage renal disease

**DOI:** 10.1371/journal.pone.0276394

**Published:** 2022-10-20

**Authors:** Dae Yong Park, Seokyung An, Jonathan M. Hanna, Stephen Y. Wang, Ana S. Cruz-Solbes, Ajar Kochar, Angela M. Lowenstern, John K. Forrest, Yousif Ahmad, Michael Cleman, Abdulla Al Damluji, Michael G. Nanna

**Affiliations:** 1 Department of Medicine, Cook County Health, Chicago, Illinois, United States of America; 2 Department of Biomedical Science, Seoul National University Graduate School, Seoul, Korea; 3 Department of Medicine, Yale School of Medicine, New Haven, Connecticut, United States of America; 4 Section of Interventional Cardiology, Brigham and Women’s Hospital, Boston, Massachusetts, United States of America; 5 Division of Cardiovascular Medicine, Vanderbilt University Medical Center, Nashville, Tennessee, United States of America; 6 Section of Cardiovascular Medicine, Yale School of Medicine, New Haven, Connecticut, United States of America; 7 Section of Interventional Cardiology, Johns Hopkins University, Baltimore, Maryland, United States of America; Ohio State University, UNITED STATES

## Abstract

**Objectives:**

We sought to examine readmission rates and predictors of hospital readmission following TAVR in patients with ESRD.

**Background:**

End-stage renal disease (ESRD) is associated with poor outcomes following transcatheter aortic valve replacement (TAVR).

**Methods:**

We assessed index hospitalizations for TAVR from the National Readmissions Database from 2017 to 2018 and used propensity scores to match those with and without ESRD. We compared 90-day readmission for any cause or cardiovascular cause. Length of stay (LOS), mortality, and cost were assessed for index hospitalizations and 90-day readmissions. Multivariable logistic regression was performed to identify predictors of 90-day readmission.

**Results:**

We identified 49,172 index hospitalizations for TAVR, including 1,219 patients with ESRD (2.5%). Patient with ESRD had higher rates of all-cause readmission (34.4% vs. 19.2%, HR 1.96, 95% CI 1.68–2.30, p<0.001) and cardiovascular readmission (13.2% vs. 7.7%, HR 1.85, 95% CI 1.44–2.38, p<0.001) at 90 days. During index hospitalization, patients with ESRD had longer length of stay (mean difference 1.9 days), increased hospital cost (mean difference $42,915), and increased in-hospital mortality (2.6% vs. 0.9%). Among those readmitted within 90 days, patients with ESRD had longer LOS and increased hospital charge, but similar in-hospital mortality. Diabetes (OR 1.86, 95% CI 1.31–2.64) and chronic pulmonary disease (OR 1.51, 95% CI 1.04–2.18) were independently associated with higher odds of 90-day readmission in patients with ESRD.

**Conclusion:**

Patients with ESRD undergoing TAVR have higher mortality and increased cost associated with their index hospitalization and are at increased risk of readmission within 90 days following TAVR.

## Introduction

Patients with end-stage renal disease (ESRD) are at risk of developing both early and rapidly progressive aortic stenosis (AS), with 10–20 years earlier onset compared with the general population [1–4]. However, they were excluded from landmark transcatheter aortic valve replacement (TAVR) trials, compromising the generalizability of the findings [5, 6]. These patients frequently have high rates of medical comorbidities, including diabetes, hypertension, malnutrition, and ischemic heart diseases, putting them at high surgical risk [7]. Lower glomerular filtration rate itself predicts adverse surgical aortic valve replacement (SAVR) postoperative outcomes [8]. Therefore, patients with ESRD are more often offered off-label TAVR than SAVR, especially with the recent U.S. Food and Drug Administration expansions of TAVR indications to include patients of any surgical risk [9, 10].

Given the emergence of TAVR as a viable alternative to SAVR for this vulnerable population, understanding TAVR outcomes in patients with ESRD is important [11]. Contemporary analyses demonstrate higher complication rates and nearly twice the 1-year mortality rate among dialysis patients undergoing TAVR, but studies following these patients from discharge after TAVR are scarce [12–14]. Readmission rates are independently associated with quality of life, healthcare costs, and increased risk of mortality [15, 16]. Therefore, we utilized the largest readmissions database in the United States to compare readmission rates in patients with and without ESRD after TAVR. We then analyzed the causes for readmission and identified factors associated with higher risk of readmission while adjusting for sociodemographic and clinical factors.

## Materials and methods

### Data source

Index hospitalizations in which a primary diagnosis of AS was treated with TAVR were identified in the National Readmissions Database (NRD) for years 2017 to 2018. A joint effort between the Healthcare Cost and Utilization Project and Agency for Health Research and Quality, the NRD records 18 million annual discharges in 30 geographically dispersed states. After applying weights, these data represent a sample of 35 million discharges [17]. The NRD longitudinally follows patients within a respective state by assigning unique identifiers to each patient; therefore, readmission to any hospital within the state will be recorded [17]. After applying discharge weights, national estimates in the United States can be generated. The NRD is publicly available with deidentified patient-level information, so the study was not under the purview of the institutional review board.

### Study population and variables

International Classification of Diseases, Tenth Revision, Procedure Coding System (ICD-10-PCS) codes 02RF37Z, 02RF38Z, 02RF3JZ, and 02RF3KZ were used to identify TAVR, and ICD-10, Clinical Modification (ICD-10-CM) codes I35.0 and I35.2 were used to identify AS. Other codes used to define comorbidities and outcomes are summarized in S1 Table. Patients 18 years and older who underwent TAVR were stratified to those with and without a secondary diagnosis of ESRD (ICD-10-CM N18.6). Index hospitalizations for transapical TAVR were excluded as it is no longer the standard of treatment [18]. Only discharges from January through September in each year were included to allow for 90 days of follow-up after discharge because patient linkage numbers do not move on to the next year. Discharges with missing census, income, and payer status were excluded. For each patient, age, sex, comorbidities, hospital characteristics, primary payer, and median income were extracted.

### Study endpoints

The primary outcomes were 90-day readmission for any cause and 90-day readmission for cardiovascular cause. Time to readmission was calculated by subtracting the time variable of subsequent hospitalization from index hospitalization. Only the first readmission within 90 days after discharge was included. Reason for readmission was attributed to the primary discharge diagnosis. Cardiovascular causes for readmission were further stratified into heart failure, myocardial infarction, stroke, arrhythmia, and other cardiovascular cause. Secondary outcomes included in-hospital mortality, length of hospital stay (LOS), and total hospital charge occurring at index hospitalizations and at readmissions within 90 days.

### Statistical analysis

Discharge weight according to stratum were applied in all analyses. Categorical and continuous variables were summarized as frequencies and means, respectively. Baseline characteristics were compared using Student’s t-test for continuous variables and chi-squared test for categorical variables. Greedy nearest neighbor propensity score matching based on demographics, comorbidities, hospital characteristics, primary payer, and median income was performed between TAVR patients with and without ESRD. Absolute standardized differences before and after matching were calculated to assess the quality of matching. Post-match differences under 10% were deemed appropriate balance [19].

Kaplan-Meier graphs over 90 days were generated and log-rank test was used to examine for statistical difference in 90-day readmissions between patients with and without ESRD. We then constructed cox proportional-hazards models with 90-day readmission as the dependent variable and ESRD status as the independent variable. Odds ratios and mean differences were calculated for categorical and continuous secondary outcomes, respectively, with respect to 90-day readmission. In addition, a multivariable logistic regression model was constructed to identify covariates associated with higher odds of readmission in patients with ESRD who underwent TAVR. Data curation, baseline comparison, and regression analyses were performed using SAS software, version 9.4 (SAS Institute, Cary, NC). Propensity score matching, survival graphs, measure of hazard ratios, and log-rank tests were conducted using *matchit*, *optmatch*, *survival*, and *survminer* packages in R version 4.0.2 (R Foundation for Statistical Computing, Vienna, Austria).

## Results

**Fig 1:**
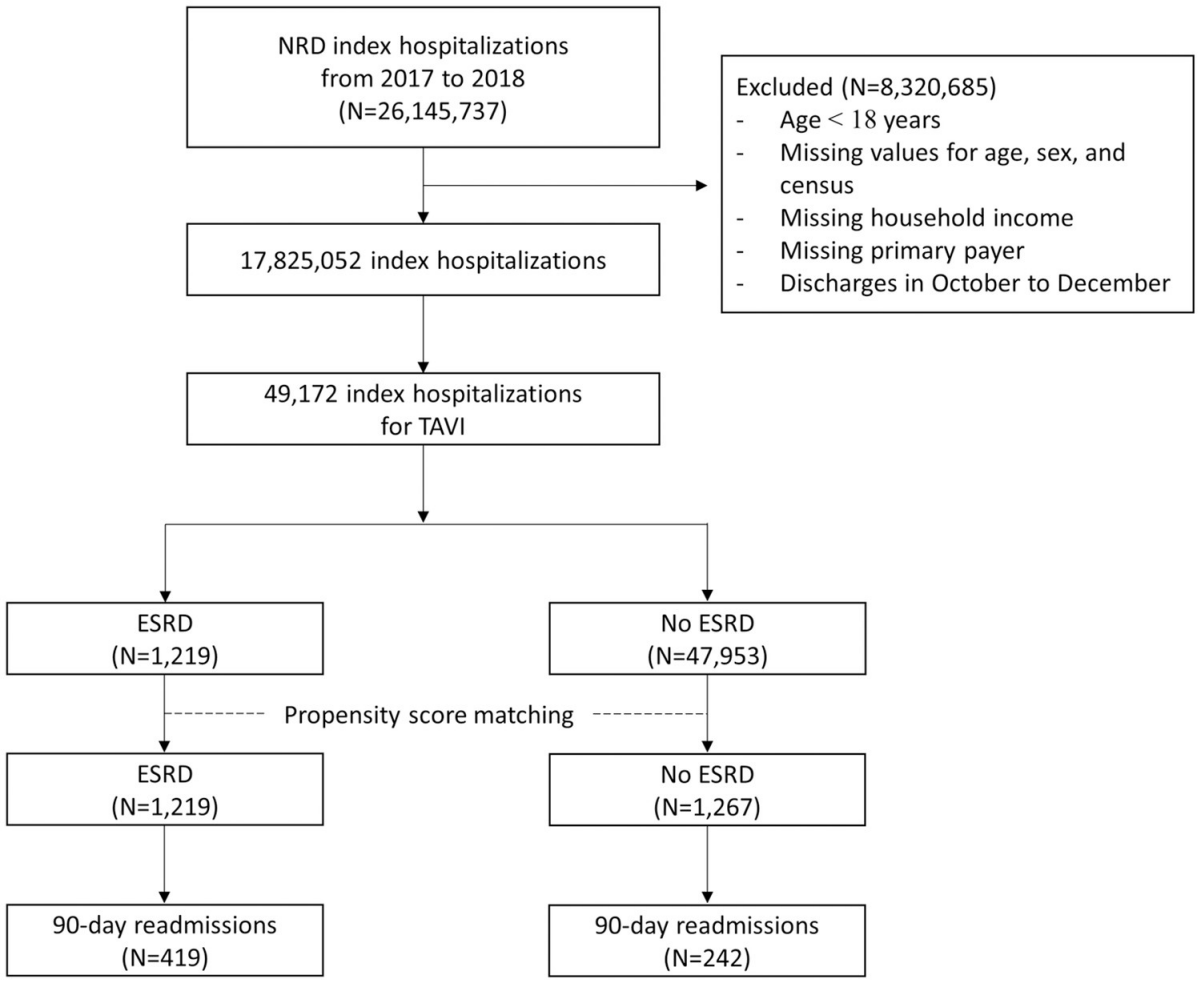
Study flow chart. Fig 1 shows the selection process of the index hospitalizations for TAVR from NRD 2017 to 2018, which are then stratified to those with and without a co-diagnosis of ESRD.

**Fig 2:**
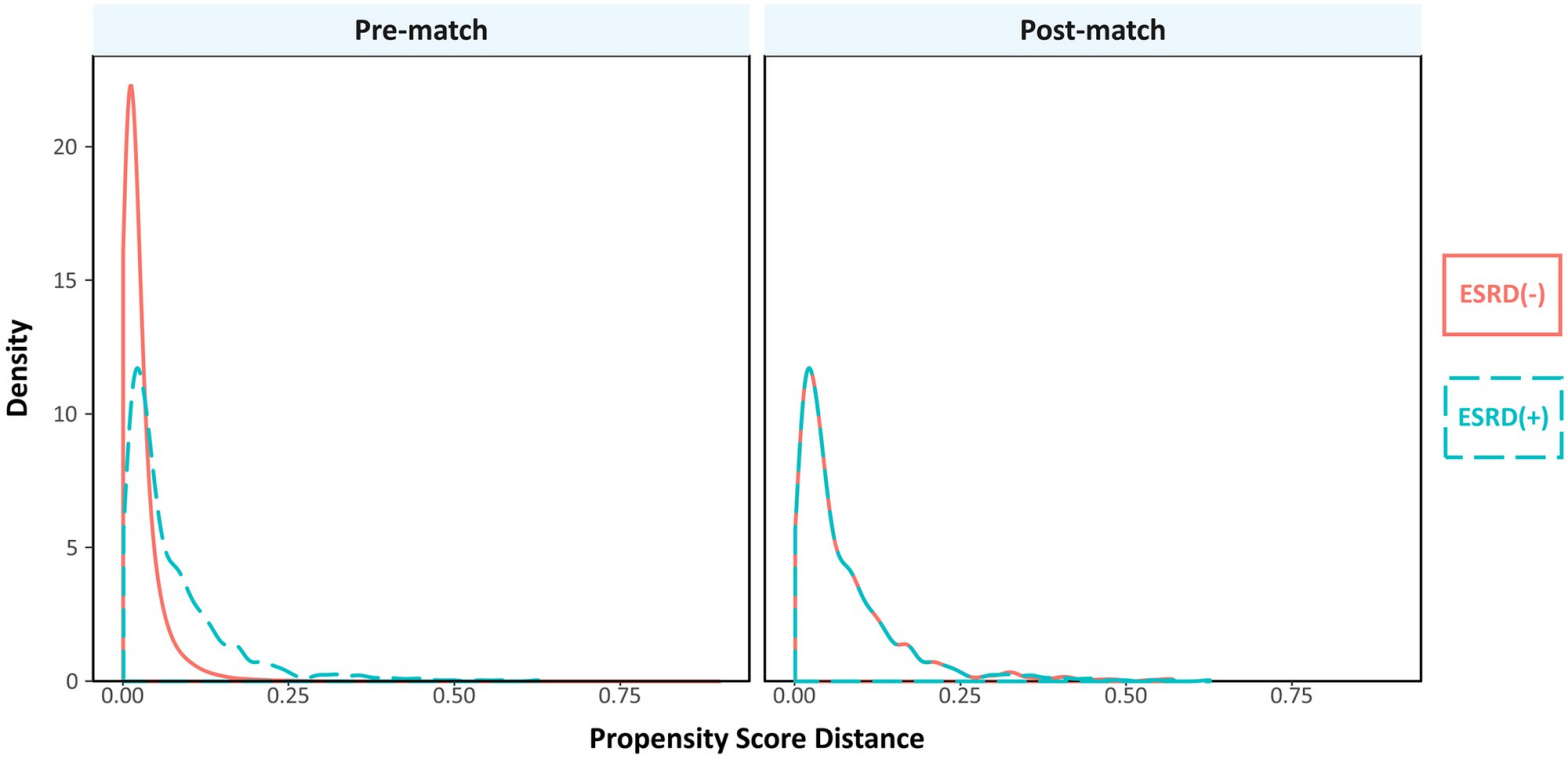
Overlap plot for propensity score. Overlap plot for the estimated density of the propensity score distances among patients undergoing TAVR with versus without co-diagnosis of ESRD.

From January to September for both 2017 and 2018, we identified 49,172 index TAVR hospitalizations (Fig 1). A total of 1,219 patients (2.5%) had ESRD. Patients with ESRD had higher rates of comorbidities, including hypertension, diabetes, heart failure, ischemic heart disease, pulmonary hypertension, liver cirrhosis, and malnutrition (Table 1). Propensity score matching produced balance of covariates between patients with and without ESRD, demonstrated by the convergence of density plots (Fig 2). Absolute standardized differences of all the covariates also remained below 10% after matching (S1 Fig). Post-match differences in baseline characteristics were also minimal (Table 1).

**Table 1:** General characteristics of index hospitalizations for TAVR with and without ESRD.

	Non-matching			Propensity score matching		
	ESRD (+)	ESRD (-)	*P*-value	ESRD (+)	ESRD (-)	*P*-value
**N (sample)**	705	27,032		705	705	
**N (weighted)**	1,219	47,953		1,219	1,267	
**Age (mean)**	74.2	80.5	<0.001	74.2	74.1	0.850
**Sex (%)**			<0.001			0.279
Male	66.5	54.4		66.5	69.2	
Female	33.5	45.6		33.5	30.8	
**Comorbidities (%)**						
Smoking	38.0	40.1	0.261	38.0	37.2	0.742
Hypertension	96.3	89.6	<0.001	96.3	96.7	0.663
Diabetes mellitus	59.6	35.7	<0.001	59.6	56.5	0.235
Hyperlipidemia	66.1	73.8	<0.001	66.1	62.7	0.182
Obesity	21.4	20.3	0.476	21.4	22.1	0.747
Heart failure	79.6	67.6	<0.001	79.6	77.2	0.272
Ischemic heart disease	74.8	69.0	0.001	74.8	73.3	0.544
Atrial fibrillation	37.9	35.4	0.183	37.9	38.4	0.826
Peripheral artery disease	7.4	7.0	0.693	7.4	7.4	1.000
Previous stroke	13.9	13.8	0.922	13.9	14.5	0.760
Previous PCI	3.7	2.3	0.016	3.7	3.8	0.889
Previous CABG	13.6	16.3	0.060	13.6	14.9	0.493
Previous pacemaker	7.2	9.1	0.090	7.2	7.9	0.615
Chronic pulmonary disease	26.8	25.9	0.588	26.8	25.1	0.466
Pulmonary embolism	0.4	0.2	0.238	0.4	0.4	1.000
Pulmonary hypertension	24.0	14.5	<0.001	24.0	25.4	0.537
Liver cirrhosis	4.0	1.6	<0.001	4.0	3.3	0.476
Deficiency anemia	4.5	4.3	0.704	4.5	3.8	0.506
Malnutrition	5.0	1.8	<0.001	5.0	5.5	0.633
**Hospital characteristics**						
**Location (%)**			0.052			0.468
Large metropolitan	68.9	63.9		68.9	71.9	
Small metropolitan	30.6	35.4		30.6	27.7	
Micropolitan	0.4	0.7		0.4	0.4	
**Bed size (%)**			0.003			0.272
Small	3.8	3.8		3.8	2.4	
Medium	16.6	22.0		16.6	17.9	
Large	79.6	74.2		79.6	79.7	
**Teaching status (%)**			0.681			0.997
Non-teaching	12.1	11.8		12.1	11.9	
Teaching	87.9	88.2		87.9	88.1	
**Primary Payer (%)**			0.738			<0.001
Medicare	90.8	91.3		90.8	84.8	
Medicaid	1.0	0.7		1.0	2.8	
Private insurance	5.7	5.9		5.7	10.6	
Self-pay	0.1	0.3		0.1	0.3	
No charge	0	0		0	0	
Others	2.4	1.8		2.4	1.4	
**Median income (%)**			0.004			0.738
Quartile 1	23.8	18.6		23.8	21.4	
Quartile 2	25.8	26.0		25.8	26.0	
Quartile 3	26.1	27.7		26.1	27.2	
Quartile 4	2.5	27.6		2.5	25.4	

Abbreviations: CABG = coronary artery bypass graft; ESRD = end-stage renal disease; IHD = ischemic heart disease; PCI = percutaneous coronary intervention; TAVR = transcatheter aortic valve replacement

At index hospitalization for TAVR, patients with and without ESRD had in-hospital mortality rate of 2.6% and 0.9%, respectively (Table 2). Co-diagnosis of ESRD was associated with higher odds of in-hospital mortality (odds ratio [OR] 3.06, 95% confidence interval [CI] 1.21–7.74, *p* = 0.02). Patients with ESRD also had longer hospital LOS (6.5 days versus 4.6 days, mean difference 1.9 days, 95% CI 1.0–2.7, *p*<0.01) and total hospital charge ($271,296 versus $228,380, mean difference $42,915, 95% CI 23,859–61,971, *p*<0.01).

**Table 2:** Comparison of outcomes in index hospitalizations of TAVR in patients with and without ESRD.

	ESRD (+)	ESRD (-)	OR (95% CI)	*P*-value
In-hospital mortality (%)	2.6	0.9	3.06 (1.21–7.74)	0.018
Length of stay (mean ± SD), days	6.5 ± 9.8	4.6 ± 6.4	1.9 (1.0–2.7)*	<0.001
Total charge (mean ± SD), $	271,296 ± 219,364	228,380 ± 136,091	42,915 (23,859–61,971)*	<0.001

*Mean difference with 95% confidence interval

Abbreviations: ESRD = end-stage renal disease; TAVR = transcatheter aortic valve replacement

**Fig 3:**
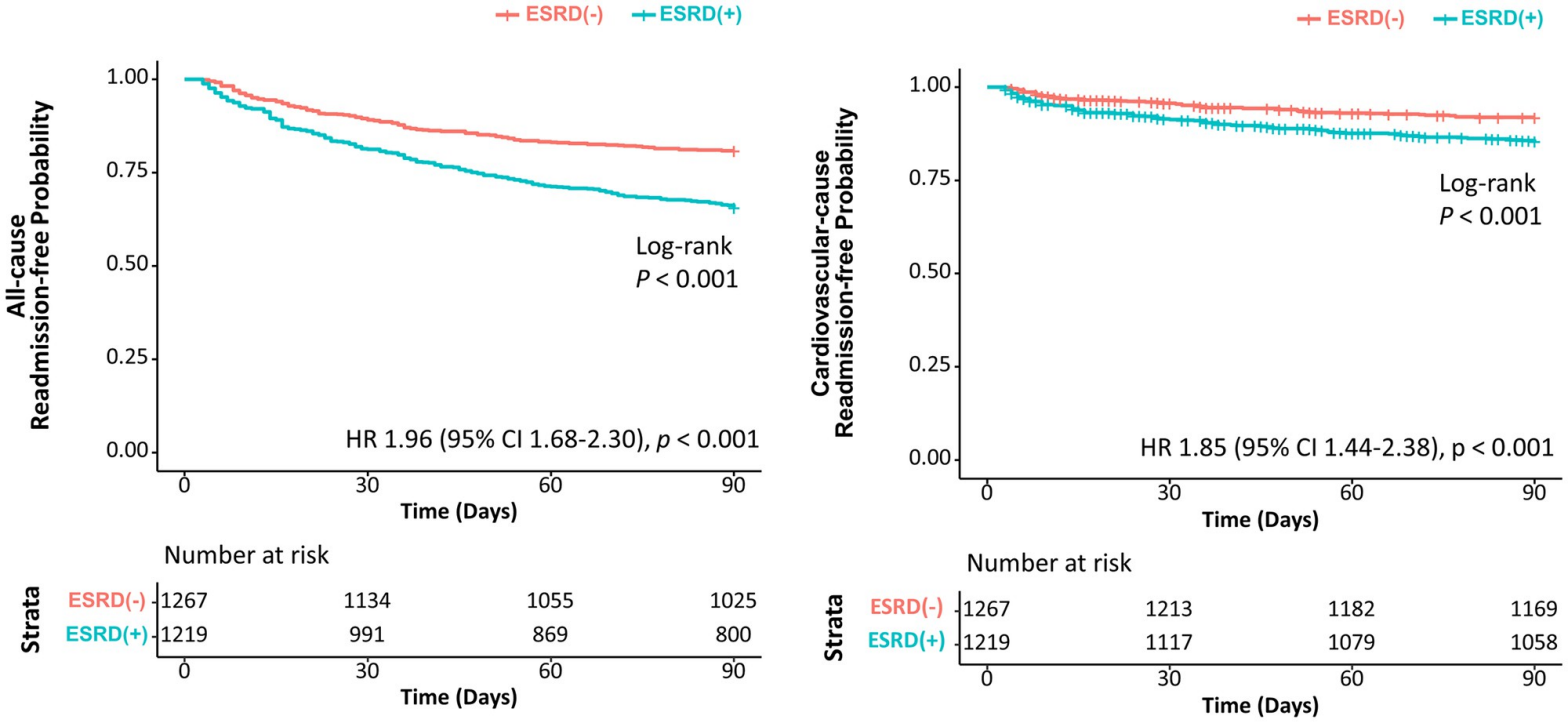
Kaplan-Meier curves of all-cause and cardiovascular readmissions after TAVR in patients with and without ESRD. Kaplan-Meier curve on the left shows the probability of being readmission-free for any cause in patients with and without ESRD over 90 days after discharge from the index hospitalization. Similarly, the Kaplan-Meier curve on the right shows the probability of being readmission-free for cardiovascular cause.

**Fig 4:**
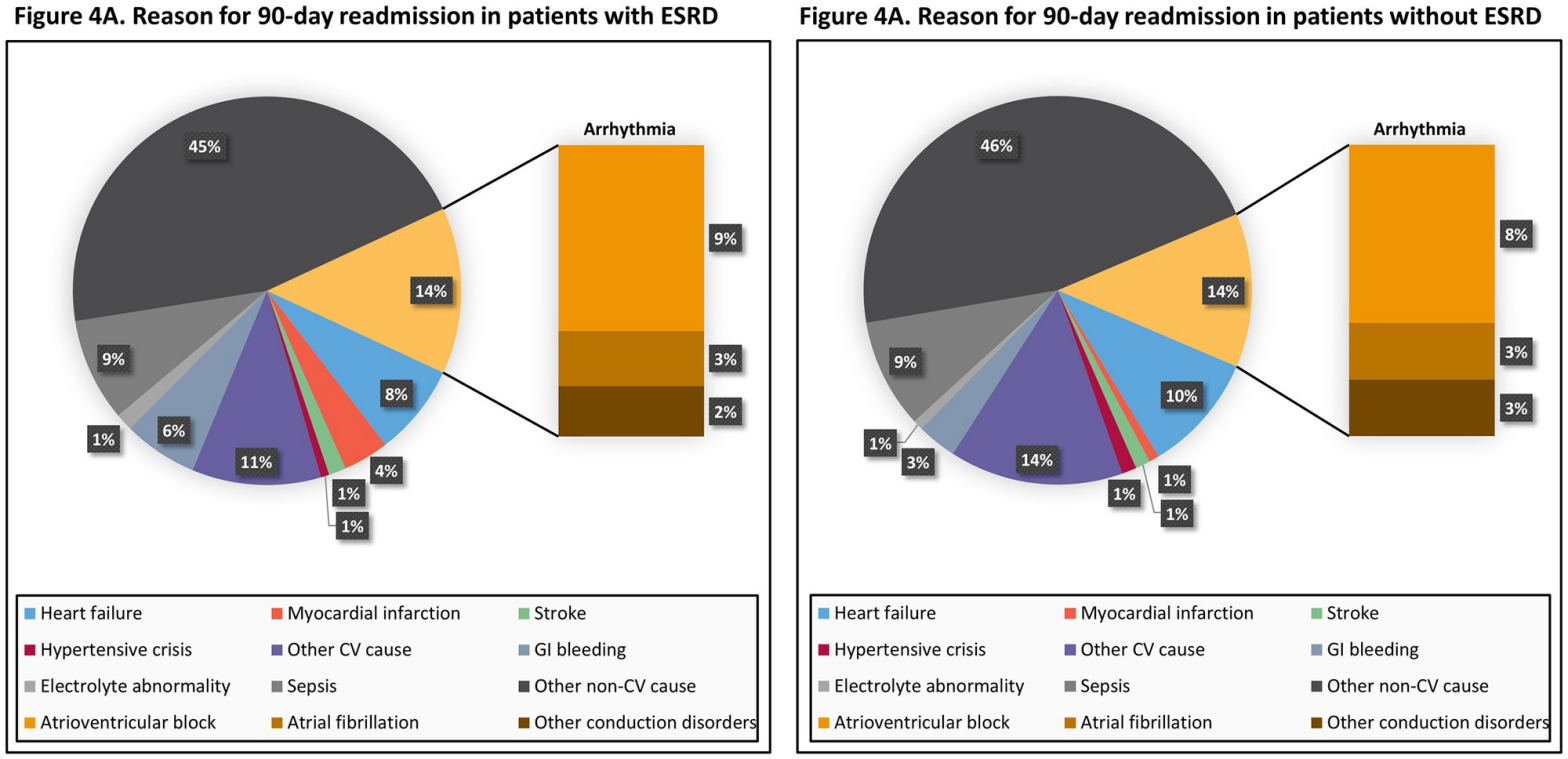
Reasons for post-TAVR readmissions in patients with and without ESRD. The pie graph on the left (Fig 4A) illustrates the percentage of different causes of 90-day readmissions in patients with ESRD. Each cause is color-coded according to the legend below the pie graph. Arrhythmia has been further divided into atrioventricular block, atrial fibrillation, and other conduction disorders. The same applies for the pie graph on the right (Fig 4B), but in patients without ESRD. Abbreviations: CV = cardiovascular; GI = gastrointestinal.

Patients with ESRD undergoing TAVR had a higher 90-day readmission rate than those without ESRD (34.4% vs 19.2%, log-rank *p*<0.001) (Fig 3). Patients with ESRD experienced almost twice the hazard of 90-day all-cause readmissions (HR 1.96, 95% CI 1.68–2.30, *p*<0.001). Patients with ESRD were also more frequently readmitted for cardiovascular causes over 90 days (13.2% vs 7.7%, log-rank *p*<0.001), with an increased hazard of 90-day cardiovascular readmission (HR 1.85, 95% CI 1.44–2.38, *p*<0.001). Sensitivity analysis examining readmissions due to atrioventricular block continued to show that patients with ESRD were at increased risk (HR 2.08, 95% CI 1.20–3.60, *p* = 0.010) (S2 Fig). In patients with ESRD, readmission diagnosis was more frequently a non-cardiovascular cause (62%), but among cardiovascular causes, arrhythmia (14%) was most common. Other causes of 90-day readmissions in patients with and without ESRD are illustrated in Fig 4.

**Fig 5:**
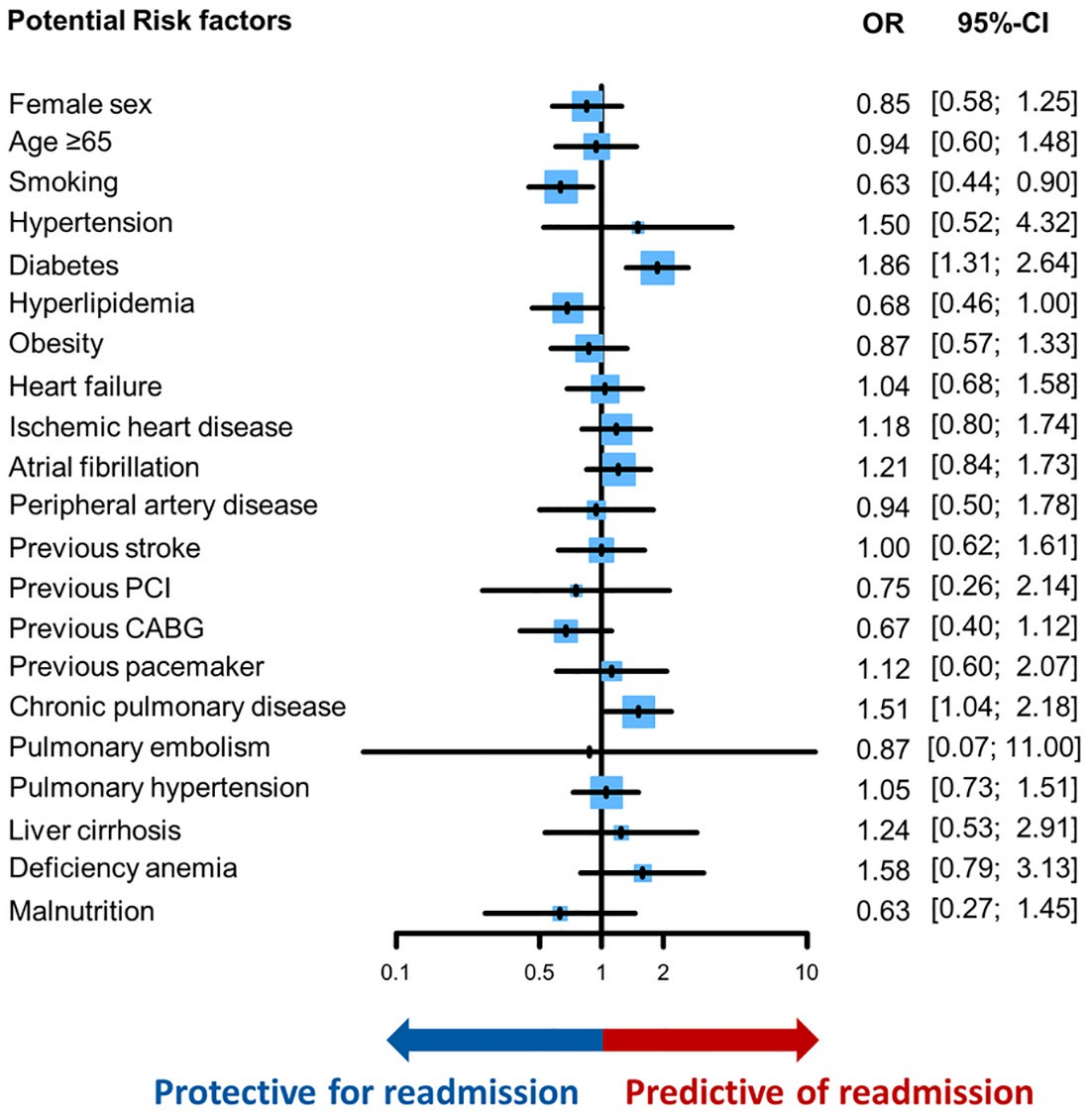
Potential risk factors for 90-day readmission after TAVR in patients with ESRD. Fig 5 shows the odds ratio of each potential risk factor associated with 90-day readmission from the multivariable logistic regression model. Odds ratio above 1 is predictive of 90-day readmission while that below 1 is protective for 90-day readmission. Each vertical line inside the blue box shows the odds ratio while the perpendicular horizontal lines show the corresponding 95% confidence interval. The size of the blue box is indirectly proportional to the size of the confidence interval.

Among patients with ESRD who underwent TAVR, diabetes (OR 1.86, 95% CI 1.31–2.64, *p*<0.01) and chronic pulmonary disease (OR 1.51, 95% CI 1.04–2.18, *p* = 0.03) were independently associated with higher odds of 90-day readmission (Fig 5). On interaction analysis, diabetes was significantly associated with ischemic heart disease (*P*-interaction = 0.04). Conversely, smoking was associated with lower odds of readmission (OR 0.63, 95% CI 0.44–0.90); the interaction between smoking and younger age was significant (*P*-interaction = 0.03). Once readmitted within 90 days, the odds of in-hospital mortality (OR 0.99, 95% CI 0.38–2.56, *p* = 0.98) between patients with and without ESRD were no longer significantly different (Table 3). However, patients with ESRD experienced longer LOS (mean difference 1.6 days, 95% 0.2–3.0, *p* = 0.02) and more expensive total hospital charge (mean difference $25,797, 95% 5,282–46,314, *p* = 0.01).

**Table 3:** Comparison of outcomes in 90-day readmissions after TAVR in patients with and without ESRD.

	ESRD (+)	ESRD (-)	OR (95% CI)	*P*-value
In-hospital mortality (%)	4.5	4.5	0.99 (0.38–2.56)	0.978
Length of stay (mean ± SD), days	6.4 ± 7.4	4.7 ± 4.9	1.6 (0.2–3.0)*	0.024
Total charge (mean ± SD), $	87,947 ± 104,238	62,149 ± 84,634	25,797 (5,282–46,314)*	0.014

*Mean difference with 95% confidence interval

Abbreviations: ESRD = end-stage renal disease; TAVR = transcatheter aortic valve replacement

## Discussion

In this large propensity-matched analysis of U.S. patients hospitalized for TAVR, patients with ESRD experienced nearly three-fold higher in-hospital mortality at index hospitalization and two-fold higher readmission rates at 90 days. Predictors of 90-day readmission included the presence of diabetes and chronic pulmonary disease. Patients with ESRD who were readmitted within 90 days had longer lengths of stay and increased cost associated with their readmission than their non-ESRD counterparts.

Our findings that patients with ESRD undergoing TAVR have increased length of stay, cost, and mortality during their index hospitalization and an increased risk of readmission are in line with the findings of previous studies demonstrating poorer outcomes in patients with impaired renal function undergoing TAVR [20, 21]. An analysis of the Society of Thoracic Surgeons/American College of Cardiology Transcatheter Valve Therapies registry demonstrated that patients with ESRD undergoing TAVR had higher in-hospital mortality and bleeding with diminished survival benefit at 1-year [14]. A meta-analysis of 10 observational studies on TAVR reported significantly higher rates of both short- and long-term mortalities and other significant complications, including major bleeding, arrhythmia, and device failure in dialysis patients compared with non-dialysis patients [21]. One study did note annually improving mid- and long-term outcomes of TAVR in 3,883 patients with ESRD, but long-term mortality at 5 years was nevertheless exceedingly high, reaching 88.8% [22]. Our study was the first to examine the outcomes of TAVR in patients with ESRD using U.S. national readmissions data, which also revealed that these patients suffered excessive rehospitalizations.

Multiple factors likely contribute to higher readmissions in patients with ESRD after TAVR. Comorbidities and functional status significantly affect the prognosis of patients with ESRD [23]. Although we achieved an appropriate balance of 19 comorbidities after propensity score-matching, the severity of these comorbidities may be worse in the ESRD group, putting them at higher risk of rehospitalization [7]. In addition, other conditions not directly included in the propensity score-match, such as mitral or tricuspid regurgitation which are known predictors of poor prognosis after TAVR, may be confounding the results [24]. However, direct consequences of ESRD itself, not limited to accelerated atherosclerosis, electrolyte imbalance, and volume overload, also likely contribute [25]. Patients with ESRD also have significant peripheral vascular calcifications, potentially leading to procedural difficulties and increased access-site complications [26, 27].

In our cohort of TAVR patients, the most common cardiovascular cause for admission was arrhythmia, followed by heart failure and myocardial infarction. The proportion of arrhythmia as a cause of readmission was similar in patients with (14%) and without (13%) ESRD, although twice as many readmissions occurred in the former. An analysis of the Netherlands Heart Registration reported serum creatinine level ≥1.13mg/dL to be significantly associated with pacemaker implantation at 30 days after TAVR, and a Swedish observational study also noted greater proportion of renal impairment in patients who had pacemakers placed after TAVR [28, 29]. Our analysis also showed that patients with ESRD were readmitted within 90 days for atrioventricular block twice (3.0% versus 1.5%) as frequently compared with patients without ESRD. Given these findings, patients with ESRD may be at higher risk of valve frame-induced conduction abnormalities leading to readmissions and pacemakers, but mechanisms are unclear and it is difficult to determine if arrhythmias leading to readmissions are directly caused by TAVR since dialysis patients have higher risk of clinically significant arrhythmias at baseline [30]. However, given these findings, patients with ESRD should be more closely monitored and followed after TAVR, and it remains to be determined if placing a temporary cardiac monitoring device in this high-risk group can lead to improved detection and outcomes.

Identifying predictors for readmission can inform potential mitigation strategies. Interestingly, smoking was associated with lower odds of readmission which may reflect the previously described “smoker’s paradox”, where smokers tend to have better outcomes than non-smokers in observational databases due to younger age and lesser burden of baseline comorbidities [31]. This hypothesis is supported by the significant interaction between smoking and younger age observed in our patient population. On the other hand, diabetes and chronic pulmonary disease were associated with higher odds of 90-day readmission. In patients with ESRD and diabetes, impaired insulin clearance and renal gluconeogenesis render patients vulnerable to low blood sugar levels as demonstrated by hypoglycemia accounting for 3.6% of all ESRD-related admissions [32]. Patients with ESRD with chronic pulmonary disease are also at risk of developing hypoxic respiratory failure from volume overload and poor underlying pulmonary reserve, putting them at risk of readmission [33]. Although dedicated studies on the impact of these comorbidities in patients with ESRD that underwent TAVR are limited, both diabetes and chronic pulmonary disease are common among patients with ESRD [23] and have been independently linked to increased risk of mortality [14, 34, 35]. Given these findings, future quality improvement efforts may be targeted at patients with ESRD undergoing TAVR, particularly those with diabetes or chronic pulmonary disease by patient education and meticulous titration of hypoglycemics or inhalers, to reduce the risk of costly readmissions.

In addition to these strategies, general efforts to reduce readmissions of patients on dialysis can be implemented as post-TAVR care since up to 50% of all readmissions are deemed avoidable in patients with ESRD [36]. Interventions performed in dialysis facilities after hospitalization, such as lowering post-dialysis weight and limiting ultrafiltration rate, may reduce readmission rates [37]. Communication and coordination between hospitals and dialysis centers with the use of post-hospital checklists, telephone case managers, and call centers have been shown to reduce all-cause readmissions [38]. Additional visits by a nephrologist, medication reconciliation, and volume optimization can also contribute to avoiding readmissions [36, 39, 40].

### Limitations

The results of our study should be interpreted in the context of several limitations. Since the NRD is a sample of the admissions in 30 states of the U.S., it may not completely represent all the readmissions in the country. However, we applied appropriate weights in all our analyses and previous studies have validated the accuracy of the NRD [41, 42]. NRD incorporates administrative data, which may contain inaccuracies and imprecise coding, but the Healthcare Cost and Utilization Project performs quality control to maintain the veracity of the database [43]. Although we assessed the presence of comorbidities, the severity of said comorbidities could not be ascertained using the NRD. However, efforts were made to produce a well-balanced match by comprehensively including all the variables in Table 1. While propensity matching achieved excellent balance of key confounders, it is impossible for account for potential imbalance across unmeasured confounders which could hypothetically contribute to observed differences in this retrospective study. Severity of AS, urgency of the presentation, ejection fraction, type of valve, and the specific indication for TAVR also could not be determined. Although we excluded transapical TAVR, isolating transfemoral TAVRs was not possible as the ICD-10-PCS codes do not further classify percutaneous TAVR approaches. We were unable to exclude valve-in-valve TAVRs given the limitations of the database. Granular data, such as the etiology of ESRD, type or renal replacement therapy, and compliance, were also unavailable. There may be a selection bias by distance and population density as patients who travel to outside states are lost and those who travel longer distances to the hospital may be subject to worse outcomes [44]. Our Kaplan-Meier curves did not account for patients who died after discharge. We used ICD-10-CM and ICD-10-PCS codes to define clinical scenarios and procedures as many validated studies have done but may nevertheless contain some misclassification bias [42, 45–47]. The NRD has also been validated by a substantial body of literature in obtaining insights into clinical questions [42, 45–47].

## Conclusion

Following TAVR, patients with ESRD have longer lengths of stay, hospital cost, and in-hospital mortality and are at higher risk of readmission at 90 days. The most common cardiovascular cause of readmission is arrhythmia, followed by heart failure. Predictors of readmission include the presence of diabetes and chronic pulmonary disease and readmitted patients with ESRD also experience longer lengths of stay and increased cost. Given the importance of TAVR in the treatment of patients with AS and ESRD [48], careful patient selection and quality improvement strategies must be targeted to mitigate the excessive risk of rehospitalization in these patients after TAVR.

## Supporting information

S1 Table List of the ICD-10 codes used. S1 Table shows the list of ICD-10 codes used in this study.
(PDF)

S1 Fig Standardized differences in baseline characteristics before and after propensity-score matching. The bar graphs show the absolute standardized difference in baseline characteristics before (red) and after (blue) propensity-score matching. The dotted vertical line demonstrates 10% absolute standardized difference.
(PDF)

S2 Fig Readmissions due to atrioventricular block after TAVR in patients with and without ESRD. The Kaplan-Meier curves show readmissions over a 90-day period due to a primary diagnosis of atrioventricular block after TAVR in patients with and without ESRD.
(PDF)
