# Supplementary material for: Readmission rates and risk factors for readmission after transcatheter aortic valve replacement in patients with end-stage renal disease
Source: PLoS One. 2022 Oct 20;17(10):e0276394. doi: 10.1371/journal.pone.0276394 (PMC9584363; doi:10.1371/journal.pone.0276394)
Supplement: S1 Table — shows the list of ICD-10 codes used in this study. (PDF) [file pone.0276394.s001.pdf]

Supplementary Table 1. List of the ICD-10 codes used

Diagnosis	ICD-10-CM Code
Aortic stenosis	I35.0, I35.2
End-stage renal disease	N18.6
Smoking	F17, T65, Z72.0, O99.33, Z78.891
Hyperlipidemia	E78
Obesity	E66, Z68.3, Z68.4
Heart failure	I09.81, I11.0, I13.0, I31.2, I50
Ischemic heart disease	I20, I21, I22, I23, I24, I25
Atrial fibrillation	I48.0, I48.1, I48.2, I48.91
Peripheral artery disease	I70
Previous stroke	I69, Z86.73
Previous PCI	Z98.61
Previous CABG	Z95.1
Previous pacemaker	Z95.0
Pulmonary embolism	I26
Pulmonary hypertension	I27.0, I27.2
Liver cirrhosis	K70.2, K70.3, K71.7, K74, K76.1, P78.81, E83.110
Deficiency anemia	D50, D51, D52, D53
Malnutrition	E43, E44, E46
Cardiovascular cause	I00-I99
Myocardial infarction	I21, I22, I23
Stroke	I63
Arrhythmia	I44, I45, I46, I48, I49
Atrioventricular block	I44.0, I44.1, I44.2, I44.3
Hypertensive crisis	I16
Electrolyte abnormality	E87
Sepsis	A40, A41, R65.10, R65.11, R65.20, R65.21, T81.12

Abbreviations: CABG = coronary artery bypass graft; PCI = percutaneous coronary intervention
