# Supplementary material for: Readmission rates and risk factors for readmission after transcatheter aortic valve replacement in patients with end-stage renal disease
Source: PLoS One. 2022 Oct 20;17(10):e0276394. doi: 10.1371/journal.pone.0276394 (PMC9584363; doi:10.1371/journal.pone.0276394)

Supplementary Figure 1. Standardized differences in baseline characteristics before and after propensity-score matching

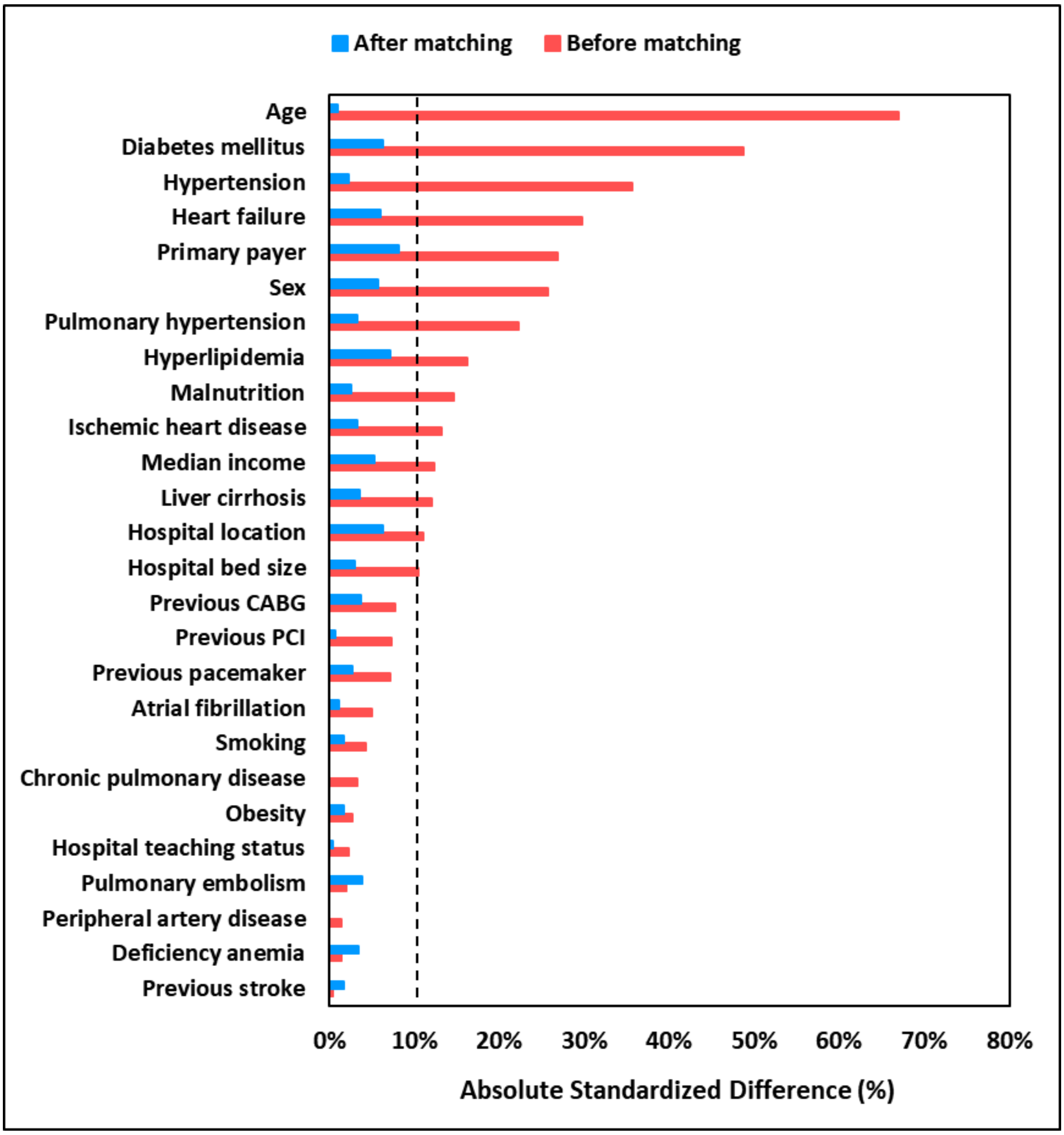

Supplement: S1 Fig — The bar graphs show the absolute standardized difference in baseline characteristics before (red) and after (blue) propensity-score matching. The dotted vertical line demonstrates 10% absolute standardized difference. (PDF) [file pone.0276394.s002.pdf]
