# Supplementary material for: Readmission rates and risk factors for readmission after transcatheter aortic valve replacement in patients with end-stage renal disease
Source: PLoS One. 2022 Oct 20;17(10):e0276394. doi: 10.1371/journal.pone.0276394 (PMC9584363; doi:10.1371/journal.pone.0276394)

Supplementary Figure 2. Readmissions due to atrioventricular block after TAVR in patients with and without ESRD

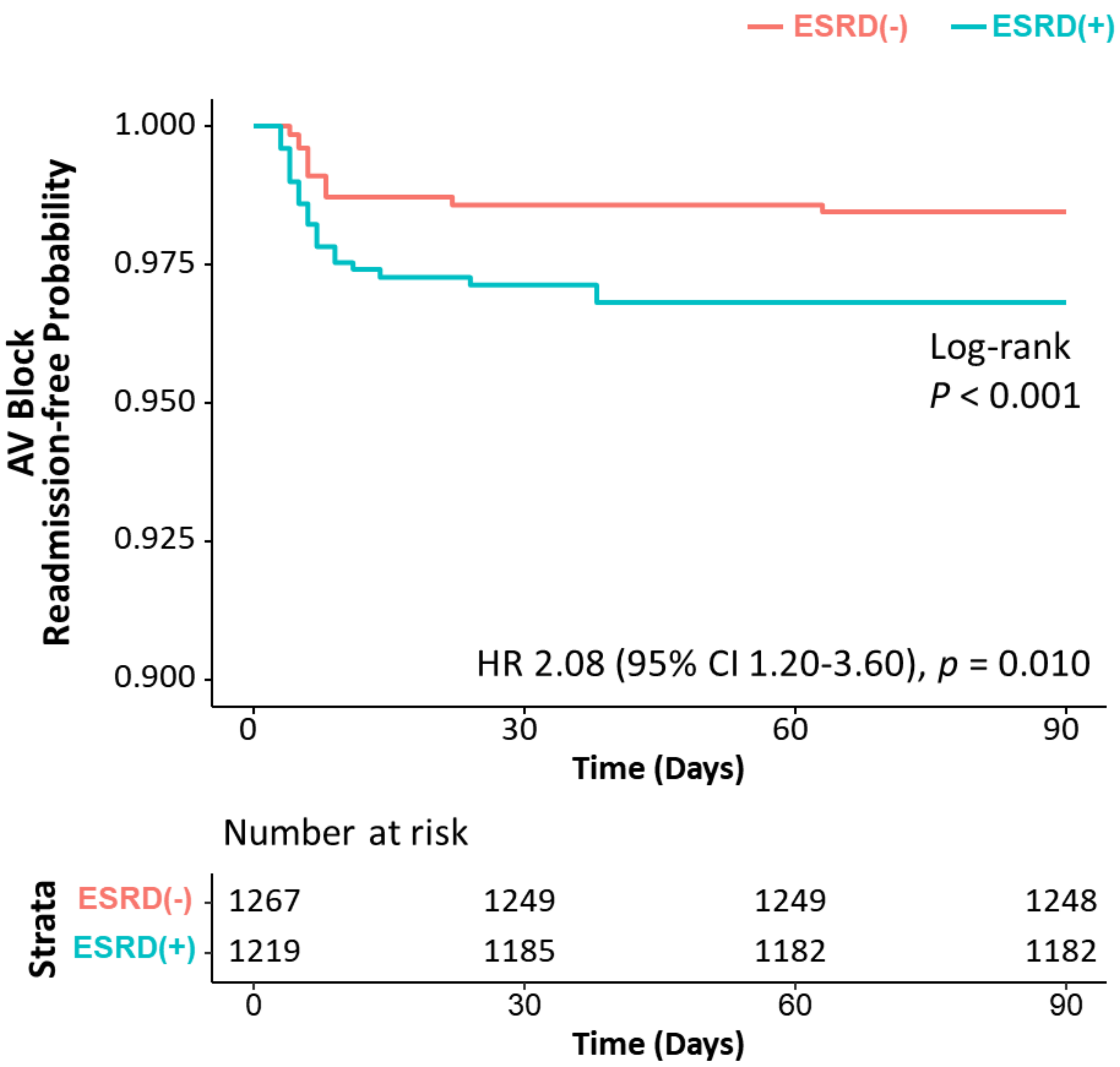

Supplement: S2 Fig — The Kaplan-Meier curves show readmissions over a 90-day period due to a primary diagnosis of atrioventricular block after TAVR in patients with and without ESRD. (PDF) [file pone.0276394.s003.pdf]
